# Bacterial topography of the upper and lower respiratory tract in pigs

**DOI:** 10.1186/s42523-023-00226-y

**Published:** 2023-01-16

**Authors:** Mattia Pirolo, Carmen Espinosa-Gongora, Antton Alberdi, Raphael Eisenhofer, Matteo Soverini, Esben Østergaard Eriksen, Ken Steen Pedersen, Luca Guardabassi

**Affiliations:** 1 Department of Veterinary and Animal Sciences, University of Copenhagen, Stigbøjlen 4, 1870 Frederiksberg C, Denmark; 2 Center for Evolutionary Hologenomics, Globe Institute, University of Copenhagen, 1353 Copenhagen, Denmark; 3 COPSAC, Copenhagen Prospective Studies On Asthma in Childhood, Herlev and Gentofte Hospital, University of Copenhagen, 2820 Gentofte, Denmark

**Keywords:** Pig, Metagenomics, Respiratory tract

## Abstract

**Background:**

Understanding the complex structures and interactions of the bacterial communities inhabiting the upper (URT) and lower (LRT) respiratory tract of pigs is at an early stage. The objective of this study was to characterize the bacterial topography of three URT (nostrils, choana, and tonsils) and LRT (proximal trachea, left caudal lobe and secondary bronchi) sites in pigs. Thirty-six post-mortem samples from six pigs were analysed by 16S rRNA gene quantification and sequencing, and the microbiota in nostrils and trachea was additionally profiled by shotgun sequencing.

**Results:**

The bacterial composition obtained by the two methods was congruent, although metagenomics recovered only a fraction of the diversity (32 metagenome-assembled genomes) due to the high proportion (85–98%) of host DNA. The highest abundance of 16S rRNA copies was observed in nostrils, followed by tonsils, trachea, bronchi, choana and lung. Bacterial richness and diversity were lower in the LRT compared to the URT. Overall, *Firmicutes* and *Proteobacteria* were identified as predominant taxa in all sample types. *Glasserella* (15.7%), *Streptococcus* (14.6%) and *Clostridium* (10.1%) were the most abundant genera but differences in microbiota composition were observed between the two tracts as well as between sampling sites within the same tract. Clear-cut differences were observed between nasal and tonsillar microbiomes (R-values 0.85–0.93), whereas bacterial communities inhabiting trachea and lung were similar (R-values 0.10–0.17). *Moraxella* and *Streptococcus* were more common in bronchial mucosal scraping than in lavage, probably because of mucosal adherence. The bacterial microbiota of the choana was less diverse than that of the nostrils and similar to the tracheal microbiota (R-value 0.24), suggesting that the posterior nasal cavity serves as the primary source of bacteria for the LRT.

**Conclusion:**

We provide new knowledge on microbiota composition and species abundance in distinct ecological niches of the pig respiratory tract. Our results shed light on the distribution of opportunistic bacterial pathogens across the respiratory tract and support the hypothesis that bacteria present in the lungs originate from the posterior nasal cavity. Due to the high abundance of host DNA, high-resolution profiling of the pig respiratory microbiota by shotgun sequencing requires methods for host DNA depletion.

**Supplementary Information:**

The online version contains supplementary material available at 10.1186/s42523-023-00226-y.

## Background

The respiratory tract is a complex organ system that is divided into upper respiratory tract (URT) and lower respiratory tract (LRT). The URT includes nose, pharynx and larynx, whereas the LRT comprises trachea, bronchia and lungs. Over the years, evidence has been accumulated in support of the close relationships between bacterial communities in the URT and LRT for humans [1, 2] and animals [3, 4]. An island model of lung biogeography has been proposed in humans [5]. According to this model, the bacterial composition of the healthy LRT microbiota is the result of the balance of microbial immigration via micro-aspiration of bacteria from the URT or inhalation of ambient air, and microbial elimination via cough, mucociliary clearance, and immune system activity [5]. Yet, no studies have compared microbiota composition and diversity between URT and LRT in pigs [6, 7].

The respiratory microbiome is crucial for the maintenance of respiratory physiology and likely plays a role in the individual susceptibility to disease pathogenesis [1, 7]. In pigs, microbiome diversity and composition in the respiratory tract likely plays an important role in regulating host immune homeostasis and preventing the porcine respiratory diseases complex (PRDC) [8]. PRDC is a major economic problem and one of the most common reasons for antimicrobial use in pig farming worldwide [9]. The aetiology of PRDC is multi-factorial and the severity of the disease depends on combination of various environmental, host and microbial factors. The disease is usually triggered by viruses such as swine influenza A virus (swIAV), porcine reproductive and respiratory syndrome virus (PRRSV) or porcine circovirus type 2 (PCV2), although some bacteria may also act as primary pathogens, namely *Actinobacillus pleuropneumoniae*, *Mycoplasma hyopneumoniae* or *Bordetella bronchiseptica* [10]. Various secondary bacterial pathogens may contribute to disease progression and severity, including lower virulence strains of *Actinobacillus suis, Glaesserella parasuis* (previously *Haemophilus parasuis*)*, Pasteurella multocida, Streptococcus suis* and *Trueperella pyogenes* [10]. Based on a recent post-mortem study of 1,658 pigs affected by PRDC [11], some pathogens seem to be correlated to specific lesions and production stages. For example, *A. pleuropneumoniae* and *M. hyopneumoniae* were significantly associated with pleuropneumonia and bronchopneumonia in fattening pigs, respectively, whereas *S. suis* was more frequently detected in pleural and pericardial lesions, mainly in weaners [11].

Interactions between URT and LRT profile of bacterial communities have been studied in various animal species [3, 4, 12], but no information is available for pigs. We recently reviewed the current knowledge of the porcine respiratory microbiome, highlighting that the majority of studies focused on the URT, and none analysed simultaneously URT and LRT microbiomes [7]. The relationship between URT and LRT microbiomes might explain individual differences in PRDC progression and severity since the URT is the primary reservoir for pathogens that reach the lungs as well as for beneficial microorganisms that may prevent pathogen’s overgrowth and dissemination towards the lungs. Understanding of the complex relationships between URT and LRT in the development of PRDC however requires a deeper knowledge of the composition of the microbiota residing in the various ecological niches of the respiratory tract in healthy pigs. Accordingly, this study was designed to characterize the topography of the respiratory tract bacterial microbiota in pigs without signs of respiratory disease.

## Materials and methods

### Sample collection and storage

Samples were collected in August 2019 from six weaned (Danish Landrace × Yorkshire) × Duroc pigs of 3–6 weeks of age weighting between 9 and 12 kg. Animals originated from two Danish indoor pig herds with weaning to 30 kg production and weaning to slaughter weight production, respectively. Both farms received weaned animals from the same herd, and they were both positive for *A. pleuropneumoniae* serotype 6 and 12 but free from *M. hyopneumoniae,* toxin-producing *P. multocida,* and PRRSV according to the Danish Specific-Pathogen Free (SPF) system*.* Pigs were euthanized (captive bolt and exsanguination) and transported on the same day to the Department of Veterinary and Animal Sciences of the University of Copenhagen. Animals were kept at 4 °C until the next morning when samples were collected post-mortem. None of the selected pigs had clinical signs or macroscopic pathological lesions of respiratory tract disease, and samples were not investigated for the occurrence of viral respiratory pathogens. Six respiratory sites were sampled for each pig. These included three URT locations (nostrils, choana and tonsils), and three LRT locations (proximal trachea, left secondary bronchi and caudal lobe). Nostrils were sampled using a single dry flocked nylon swab (COPAN Diagnostics) which was introduced in each nostril for *ca*. 2–3 cm and rotated 5 times. The mucosa of the nasal concha was swabbed after aseptically removing the nasal septum. The jaw was aseptically dissected and both tonsils were swabbed with a single swab. The trachea and lungs were then removed, and an incision was made in the cranial part of the trachea where a swab was introduced approximately 5 cm caudally to the larynx and rotated 5 times. The caudal left lung lobe was isolated, and 2 ml of saline was introduced in the lobe and immediately aspirated with a Pasteur pipette. The lavage was repeated 5 times and *ca*. 7 ml of saline was recovered in a sterile tube. Mucosa from the secondary left bronchi was scraped with a sterile scalpel and placed in a sterile cryotube. After collection, all samples were immediately placed in ice. The tip of each swab was cut and placed in a sterile cryotube. After centrifugation at 5000 rpm for 10 min, the supernatant of each bronchoalveolar lavage was discarded except for 0.5 ml. The sample was then vortexed to resuspend the pellet and transferred in a sterile cryotube. All samples were stored at − 20 °C until DNA extraction.

### DNA extraction and bacterial 16S rRNA gene quantification by qPCR

Total DNA from swabs, tissue and lung lavage was extracted using the DNeasy PowerSoil Kit (Qiagen) according to the manufacturer’s instructions. Sterile swabs and saline were included as negative control in the DNA extraction protocol.

Total bacteria were estimated by quantitative PCR (qPCR) using 16S rRNA primers 338F (ACTCCTACGGGAGGCAG) and 530R (GTATTACCGCGGCTGCTG), as previously described [13]. Real-time PCR assay was performed on the LightCycler 96 System (Roche Life Science) in 20 µl reactions with FastStart Essential DNA Green Master mix (Roche Life Science) with the additions of each primer at a concentration of 0.5 µM. The cycling conditions were as follows: 2 min at 95 °C; 40 cycles of 20 s at 95 °C and 60 s at 61 °C; and a melt curve step from 60 to 95 °C.

A qPCR standard curve was created with tenfold dilutions of the full-length 16S rRNA gene amplified from *Escherichia coli* ATCC 25,922 using primers 8F (AGAGTTTGATCCTGGCTCAG) and 1492R (GGTTACCTTGTTACGACTT) and the DreamTaq Green PCR Master Mix (ThermoFisher Scientific). The PCR was carried out in the ProFlex PCR System (Applied Biosystems) under the following conditions: 2 min at 95 °C; 30 cycles of 30 s at 95 °C, 30 s at 52 °C, and 90 s at 72 °C; 5 min at 72 °C. The PCR products were purified using the GeneJET PCR Purification Kit (ThermoFisher Scientific) according to the manufacturer’s instructions. The PCR products were visualised on a 1% agarose gel containing ethidium bromide and GeneRuler 1 kb Plus DNA Ladder (ThermoFisher Scientific) to ensure fragment lengths approximating 1484 bp. DNA concentration was determined by the Qubit quantification system (Life Technologies). The 16S rRNA gene copy number was calculated using the equation: copy number = (*C*/*X*) × 0.912 × 10^12^ with C the DNA concentration measured (ng/µl) and X the PCR fragment length (bp).

The number of target copies in each sample was then calculated using the equation: copy number = [10^(− 1/*S*)]^(*I* − Ct), where S is the slope of the log-linear part of the standard curve, I the intercept of the standard curve, and Ct is the cycle threshold of the sample. Number of copies was expressed as a function of grams of DNA.

### Libraries preparation and sequencing

Partial 16S rRNA gene sequences were amplified using the Quick-16S NGS Library Prep Kit (Zymo Research), which target the V3-V4 region of the 16S rRNA gene. Amplification was carried out by using the LightCycler 96 System (Roche Life Science). Illumina adapters were added to the partial 16S rRNA gene-specific amplicons, which were further processed using the Quick-16S NGS Library Prep Kit (Zymo Research). Amplicons were pooled in equimolar ratios and then purified with the Select-a-Size MagBead (Zymo Research). DNA concentration of the sequence library was determined by the Qubit quantification system (Life Technologies). Each step of the library preparation was performed using ZymoBIOMICS Dnase/Rnase Free Water (Zymo Research). A negative control was sequenced to verify that contaminations did not occur during the amplification and sequencing phases. Furthermore, sequencing performance was validated using a synthetic mock community of eight known organisms (ZymoBIOMICS Microbial Community DNA Standard, Zymo Research). Sequencing was performed on an Illumina MiSeq platform (2 × 300 bp paired-end reads) using the MiSeq Reagent Kit v3 (600 cycles; Illumina), according to manufacturer’s instructions.

The genomic DNA from nostrils and trachea was randomly sheared into fragments of around 350 bp for shotgun sequencing. The fragmented DNA was used for library construction using the NEBNext Ultra Library Prep Kit for Illumina (New England Biolabs). The prepared DNA libraries were evaluated using Qubit quantitation system (Life Technologies) and Bioanalyzer 2100 (Agilent Technologies) for the fragment size distribution. Sequencing by Illumina HiSeq (2 × 150 bp paired-end reads) was outsourced to Novogene (Cambridge, United Kingdom).

All sequencing data were deposited to the NCBI Sequence Read Archive (SRA) under BioProject PRJNA825695.

### Bioinformatics analysis

16S rRNA sequencing data were processed using DADA2 v1.14.1 [14] as implemented in R v3.6.1. Optimal filtering and trimming parameters were identified using FIGARO v3.0 [15]. A taxonomy table was assembled by assigning taxonomy to each amplicon sequence variant (ASV) using the Silva taxonomic database v.138.1 for DADA2 [16]. Potential contaminants were identified using control samples (n = 3) and removed using decontam v.1.12.0 [17]. Sequences matching mitochondria or chloroplast were also removed, along with any sequences not assigned to Bacteria. A phyloseq object was constructed from the ASV and taxonomy tables in R using phyloseq v1.30.0 [18] for subsequent analysis.

Shotgun metagenomic data were processed using a custom pipeline built on Snakemake [19]. Paired end reads were quality controlled using fastp v0.20.1 [20], with the following options: -trim_poly_g, -trim_poly_x, -n_base_limit 5, -qualified_quality_phred 20, -length_required 35. Processed reads were then mapped to the *Sus scrofa* (Sscrofa11.1) reference genome assembly using Bowtie2 [21] and samtools [22], with default settings. Reads that did not align to the host genome from all samples were co-assembled using metaSPAdes [23], with the following kmer sizes: 21, 29, 39, 59, 79, 99, 119. Co-assembly contigs shorter than 1500 bp were removed. Each sample’s reads were then mapped to the co-assembly using Bowtie2, and the resulting BAMs were used as input to the MetaWRAP binning module [24]. The resulting bins were automatically refined using MetaWRAP’s bin_refinement module, with a minimum completeness score of 70% and minimum contamination score of 10%. Refined bins were dereplicated with dRep [25] into clusters with > 98% average nucleotide identity (ANI). The non-host quality controlled reads were then mapped against this dereplicated metagenome-assembled genome (MAG) catalogue as above. BAMs were then profiled using CoverM (https://github.com/wwood/CoverM) to create the final sample count table. Dereplicated MAGs were also taxonomically annotated using GTDB-tk [26]. Finally, MAGs were functionally annotated using the DRAM pipeline [27], and screened for the presence of resistance determinants using ABRicate v1.0.1 against the ResFinder database, and alignment results with identity scores greater than 95% were selected as positive matches. Multi-locus sequence typing (MLST) was performed using mlst (https://github.com/tseemann/mlst) using the PubMLST database (https://pubmlst.org/).

*α*-diversity indexes (Shannon and Chao1) were calculated using vegan v.2.5.7 on ASVs after normalization by scaling with ranked subsampling (SRS) [28] at 1600 sequences per sample. Multiple comparison of α-diversity indexes, 16S rRNA gene copies and relative abundance of PRDC pathogen was performed using the Wilcoxon Rank Sum test and *p*-values were corrected for multiple comparisons using Holm’s correction (i.e., *q*-values).

*β*-diversity was assessed using the Bray–Curtis dissimilarity metric and visualized using a non-metric multi-dimensional scaling (NMDS) plot using the phyloseq package. Differences between site was investigated by permutational multivariate analysis of variance (PERMANOVA) using the Adonis function from the vegan package. To evaluate compositional similarities between different sampling locations, an individual analysis of similarity (ANOSIM) using the Bray–Curtis dissimilarity index was performed for each pairwise comparison and all *p*-values were adjusted for multiple comparisons using the Benjamini-Hochberg’s correction. ANOSIM is a permutation-based ANOVA-like test that compare differences between groups [29]. The test provides R-value associated with the similarity between the tested groups: groups with an R-value close to 0 are highly similar, while groups with an R-value close to 1 can be clearly discriminated [29].

Shared and unique genera between each site were calculated with jvenn [30] on ASVs that were agglomerated at the genus level (i.e., all ASVs classified as the same genus were combined) and visualized as intersection plot using UpSetR v.1.4.0 [31]. Differential abundance analysis was performed using DESeq2 on and contrasts were corrected for multiple comparisons using the Benjamini-Hochberg’s correction. Only ASVs with *q*-values < 0.01, estimated fold change > 6 or < − 6, and estimated base mean > 60 were considered significantly differentially abundant.

## Results

### 16S rRNA profiling of the respiratory bacterial community

The mean of 16S rRNA copies detected by qPCR was significantly higher in URT samples compared to LRT samples (7.0 vs*.* 6.0 log_10_ copies/g, *p* = 0.00024). This difference was driven by nasal and tonsillar samples, which showed the highest abundance of 16S rRNA copies, 7.2 and 7.1 log_10_ copies/g, respectively. The numbers of copies in samples from trachea (6.2 log_10_ copies/g), bronchi (6.0 log_10_ copies/g) and choana (5.8 log_10_ copies/g) were similar, and the lowest copy number was observed in lung aspirate (4.6 log_10_ copies/g) (Additional file 1: Fig. S1). Pairwise Wilcoxon rank-sum test results comparing 16S rRNA copies are provided in Additional file 1: Table S1.

**Fig. 1:**
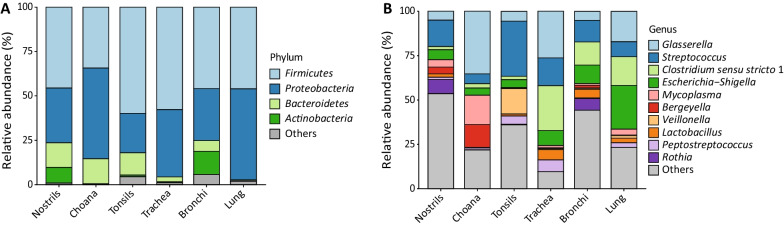
Relative composition of bacteria at phylum (**A**) and genus (**B**) level between different sampling locations. The top 10 most abundant genera are displayed

16S rRNA gene sequencing generated a total of 8,151,070 paired-end reads across all samples (range 67,355–663,461 reads), which were clustered in 2,679 ASVs after quality-filtering. The distributions of the dominant phyla and genera identify by 16S rRNA gene sequencing across all sampling locations are presented in Fig. 1A and B. Firmicutes, Proteobacteria, Bacteroidetes and Actinobacteria were identified in all locations with an overall relative abundance of 48.2%, 37.1%, 8.3% and 3.9%, respectively (Table 1). Although highly variable in abundance (Fig. 1B), 55 genera occurred in all sampling sites (Additional file 1: Fig. S2). Overall, the top 10 most abundant genera were *Glasserella* (15.7%), *Streptococcus* (14.6%), *Clostridium *sensu stricto 1 (10.1%), *Escherichia-Shigella* (9.6%), *Mycoplasma* (4.4%), *Bergeyella* (3.2%), *Veillonella* (2.8%), *Lactobacillus* (2.8%), *Peptostreptococcus* (2.7%) and *Rothia* (2.5%) (Fig. 1B and Table 1). Unclassified genera accounted for a mean relative abundance of 1.4% per sample (range 0.02–18.0%). Nostrils had the highest number of unique bacterial genera (n = 90), followed by bronchi (n = 24), tonsils (n = 21), choana (n = 21), trachea (n = 8) and lung (n = 8) (Additional file 1: Fig. S2).

**Table 1:** Mean relative abundance of bacteria present at ≥ 1% at phylum, genus and species level across all sampling locations

Phylum (%)	Genus (%)	Species (%)
*Firmicutes* (48.2)	*Streptococcus* (14.6)	*S. suis* (4.2), *S. porci* (1.9)
	*Clostridium *sensu stricto 1 (10.1)	*C. perfringens* (9.3)
	*Mycoplasma* (4.4)	*M. hyorhinis* (4.2)
	*Veillonella* (2.8)	*V. caviae* (2.5)
	*Lactobacillus* (2.8)	*L. amylovorus* (1.9)
	*Peptostreptococcus* (2.7)	
	*Paeniclostridium* (2.1)	*P. sordellii* (2.1)
	*Weissella* (2.0)	*W. cibaria* (1.7)
	*Staphylococcus* (1.5)	
*Proteobacteria* (37.1)	*Glasserella* (15.7)	*G. indolica* (15.7)
	*Escherichia-Shigella* (9.6)	
	*Mannheimia* (2.4)	*M. varigena* (2.4)
	*Acinetobacter* (1.8)	*A. lwoffii* (1.2)
	*Pasteurella* (1.5)	*P. multocida* (1.4)
	*Klebsiella* (1.2)	
*Bacteroidetes* (8.3)	*Bergeyella* (3.2)	*B. zoohelcum* (3.1)
	*Empedobacter* (1.6)	*E. brevis* (1.6)
	*Prevotella* (1.2)	
*Actinobacteria* (3.9)	*Rothia* (2.5)	*R. nasimurium* (2.4)

**Fig. 2:**
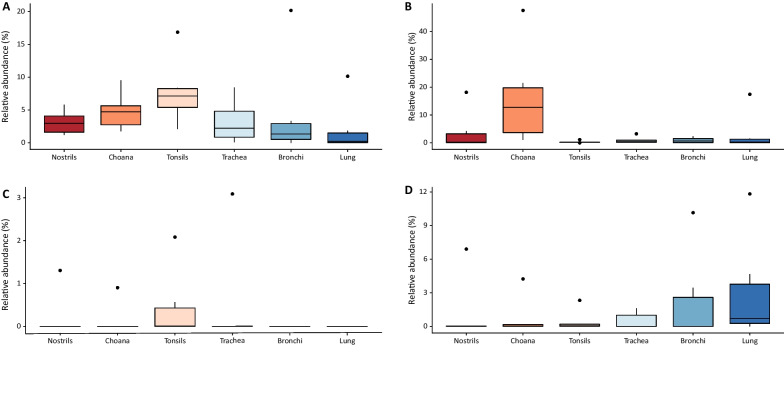
Relative abundance of PRDC pathogens between different sampling locations. **A** *Streptococcus suis*, **B** *Mycoplasma hyorhinis*, **C** *Trueperella pyogenes* and **D** *Pasteurella multocida*

On average, more than 70% of ASVs per sample could be classified at species level, among which *Glaesserella indolica* (15.7%) and *Clostridium perfringens* (9.3%) were the most abundant species identified in the dataset (Table 1). Amongst opportunistic PRDC pathogens, *S. suis* was present in all sampling locations (Fig. 2A), whereas *M. hyorhinis* and *T. pyogenes* were prevalent in choana and tonsils, respectively (Fig. 2B and C), and *P. multocida* was mostly present in bronchial and lung samples (Fig. 2D). However, no significant differences were observed in the relative abundance of PRDC pathogens between sites.

### Variation in diversity between sites

**Fig. 3:**
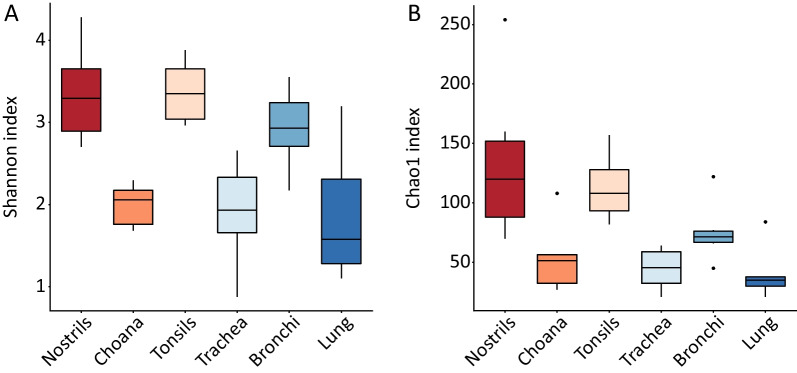
Comparison of α-diversity indexes between sampling sites. Box-plot of α-diversity calculated with the Shannon **A** and Chao1 **B** diversity indexes

Shannon and Chao1 diversity indexes differed between sample sites (Fig. 3A and B). The mean diversity index (DI) was significantly higher in URT than in LRT samples using both Shannon (2.9 vs*.* 2.2, *p* = 0.02) and Chao1 (100.2 vs. 53.4, *p* = 0.004) indexes. Nostrils, tonsils and bronchi showed comparable levels of Shannon diversity, which was significantly higher than the three other sites (Fig. 3A). A similar pattern was observed for the Chao1 index (Fig. 3B) but limited to nostrils and tonsils. Pairwise Wilcoxon rank-sum test results comparing *α*-diversity indexes are provided in Additional file 1: Table S1.

### Community analysis of the URT and LRT microbiome

**Fig. 4:**
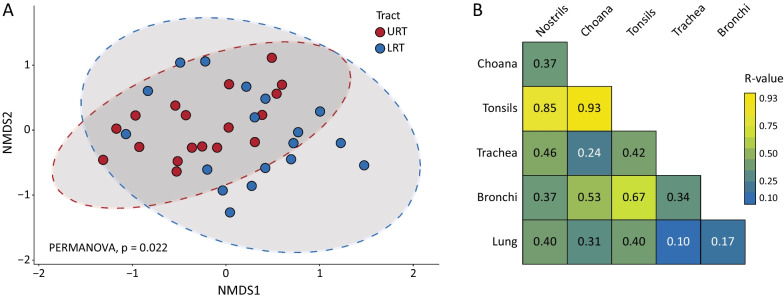
Beta-diversity analysis between URT and LRT samples. **A** Two-dimensional non-metric multidimensional scaling (nMDS) plot based on the Bray–Curtis dissimilarity matrix was used to simultaneously visualise individual samples (dots) originating from URT (nostrils, choana and tonsils) and LRT (trachea, bronchi and lung). Sample clustering of the two respiratory tract was significantly different (PERMANOVA, *p* = 0.022). **B** Pairwise analysis of similarities (ANOSIM) results between sampling sites. R-values indicate the strength of the differences, where 1 is the strongest and 0 is weakest

A nMDS plot based on the Bray–Curtis dissimilarity matrix was used to visualise differences in the community structure between URT and LRT samples (Fig. 4A). This showed that the URT and LRT microbiome significantly differed in community composition (PERMANOVA, *p* = 0.022). Visual inspection of the plot revealed that URT samples formed a discrete cluster within the LRT samples, which were scattered within the plot (Fig. 4A). Pairwise ANOSIM analysis revealed that samples collected from choana and nostrils were dissimilar to those collected from tonsils (R-values 0.85–0.93, *q*-values < 0.05) (Fig. 4B). Beta-diversity analysis at animal level confirmed these results, with tonsils samples from each pig forming a discrete cluster that was separated from nasal samples (Additional file 1: Fig. S3A). The lung microbiota was more similar to the tracheal and bronchial microbiotas (R-values 0.10–0.17 and *q*-values 0.930–0.763) than to those residing in the URT, although a certain degree of similarity was observed between the tracheal and choanal microbiotas (R-value 0.24 and *q*-values 0.084). At individual level, the three LRT microbiotas clustered together according to animal ID in all but one pig (Additional file 1: Fig. S3B). R-values and statistic results for all pairwise comparisons are provided in Additional file 1: Table S2.

**Fig. 5:**
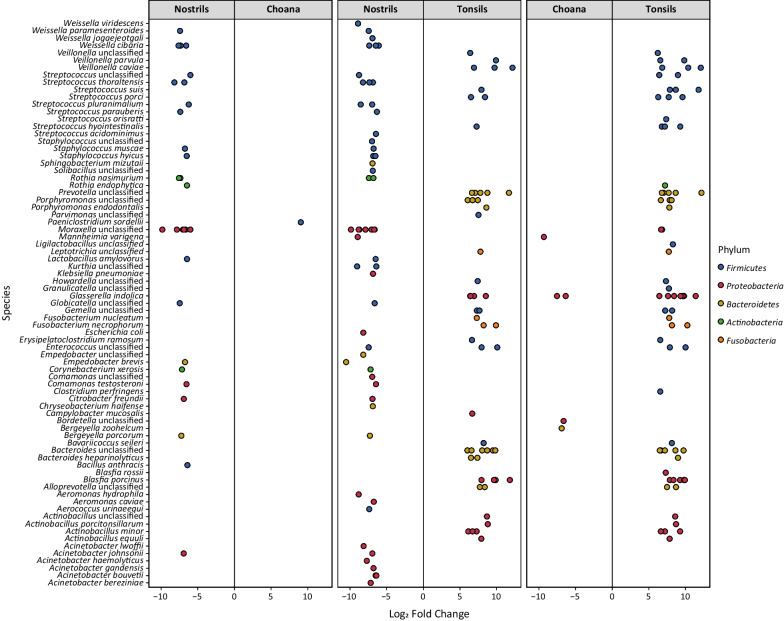
Differential abundance analysis between URT sites. ASVs were identified to be of significantly differential abundance by DESeq2. Only ASVs with *q*-values (adjusted *p*-values) < 0.01, estimated fold change > 6 or < − 6, and estimated base mean > 60 were considered significantly differentially abundant and included in the plot

To substantiate dissimilarities observed between URT locations (Fig. 4B), a detailed investigation of ASVs associated to URT sites was carried out using DESeq2. By applying highly stringent parameters (*q*-values < 0.01, estimated log_2_ fold change > 6 or < − 6, and estimated base mean > 60), 139 differentially abundant ASVs between sampling locations were identified in all pairwise comparison (Fig. 5). Among them, ASVs assigned to *Moraxella*, *Weissella*, *Acinetobacter*, *Rothia* and *Staphylococcus* were associated with nostrils, whereas those assigned to *Actinobacillus*, *Bacteroides*, *Veillonella* and *Prevotella* were associated with tonsils (Fig. 5). Distribution of ASVs assigned to the genus *Streptococcus* followed a species-specific pattern, with *Streptococcus thoraltensis*, *Streptococcus pluranimalium* and *Streptococcus acidominimus* linked to nostrils, and *S. suis*, *Streptococcus porci* and *Streptococcus hyointestinalis* associated with tonsils (Fig. 5).

When performed on LRT sites, DESeq2 analysis identified a lower number (n = 41) of differentially abundant ASVs compared to the URT analysis (Additional file 1: Fig. S4). This confirmed the close similarity between LRT locations, with only few patterns of ASVs associated with a specific location, namely *Moraxella* and *Streptococcus* that were linked to the bronchial samples (Additional file 1: Fig. S4).

### MAGs of nasal and tracheal samples

Shotgun sequencing of the 12 samples from nostrils and trachea generated 130 Gb of data. Following read trimming, samples yielded an average of 66 million paired-end reads per sample (range 56–94 million paired-end reads). However, around 95% of reads per sample mapped to the host (range 85.4–98.4%), leading to an average of 3.5 million non-host reads (range 1–9 million reads). Thirty-two MAGs were obtained from co-assembly of non-host reads, of which 21 showed a CheckM completeness > 90% and contamination < 5%. The presence of the 23S, 16S, and 5S rRNA genes and at least 18 tRNAs was confirmed in two highly complete MAGs (MAG 28 and MAG 20), which would therefore be defined as high-quality draft genomes [32]. The assembly quality statistics of the 32 genomes are summarized in Additional file 1: Table S3. All 32 MAGs were taxonomically annotated to at least family level using the Genome Taxonomy Database (GTDB). In agreement with 16S rRNA data, *Proteobacteria* (n = 14 MAGs) and *Firmicutes* (n = 13 MAGs) were predominantly identified, with *Bacteroidetes* and *Actinobacteria* accounting for a low proportion of MAGs (n = 4 and 1, respectively). Among MAGs classified at genus level (n = 30), *Acinetobacter* (n = 4), *Moraxella* (n = 4) and *Streptococcus* (n = 3) were predominant in the dataset (Additional file 1: Table S3). MAGs assigned to PRDC secondary pathogens, including *G. parasuis*, *M. hyorhinis*, *S. suis* and *P. multocida*, were also identified (Additional file 1: Table S3)*.* Notably, seven genomes shared < 95% ANI with the reference genomes of GTDB and therefore may represent uncultivated bacteria novel taxa, including three unclassified species assigned to the *Moraxella* genus (Additional file 1: Table S3). MAG ID 13, which was taxonomically assigned to *Shigella flexneri* (which belongs to the same genomospecies *E. coli*) was identified with high frequencies in both nasal and tracheal samples (9/12 samples, mean relative abundance of 45.7%), possibly due to contamination, and was therefore excluded from analysis.

**Fig. 6:**
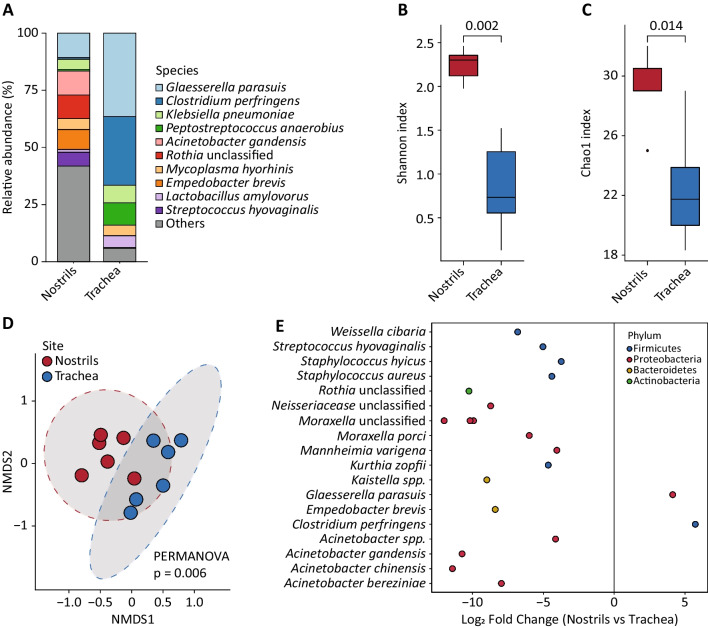
Comparison of nostril and tracheal samples based on the MAG count table. **A** Relative abundance of the top 10 abundant MAGs. **B** Shannon and **C** Chao1 diversity indexes. Groups were compared with the Wilcoxon Rank Sum test. **D** Two-dimensional non-metric multidimensional scaling (nMDS) plot based on the Bray–Curtis dissimilarity matrix of nasal and tracheal samples. Sample clustering of the two respiratory tract was significantly different (PERMANOVA, *p* = 0.006). **E** Differential abundance analysis between nasal and tracheal samples based on DESeq2. Only MAGs with *q*-values (adjusted *p*-values) < 0.01, estimated fold change > 3 or < -3, and estimated base mean > 60 were considered significantly differentially abundant and included in the plot

Comparison of the most abundant genera identified by 16S rRNA and shotgun are provided in Table 2. Community composition inferred from shotgun data showed that *G. parasuis* was the predominant species identified in both nostrils and trachea, with a relative abundance of 10.6% and 36.5%, respectively (Fig. 6A). *Acinetobacter gandensis* (10.5%), *Rothia* unclassified (10.3%) and *Empedobacter brevis* (8.9%) were highly abundant in the nose, whereas *C. perfringens* (30.0%) and *Peptostreptococcus anaerobius* (9.8%) were found in tracheal samples (Fig. 6A).

**Table 2:** Relative abundance (%) of the most abundant genera identified in nostrils and trachea by shotgun and 16S rRNA sequencing

Genus	Anterior nares		Trachea	
	Shotgun	16S	Shotgun	16S
*Glaesserella*	10.63	4.98	36.47	26.28
*Clostridium*	0.83	1.76	30.03	25.31
*Acinetobacter*	18.01	6.23	0.04	0
*Klebsiella*	4.40	2.24	7.69	0.69
*Peptostreptococcus*	0.72	1.16	9.81	6.61
*Rothia*	10.33	8.03	0	0
*Mycoplasma*	4.73	4.19	4.58	1.22
*Streptococcus*	7.85	14.93	1.24	15.68
*Empedobacter*	8.78	6.84	0.02	0
*Lactobacillus*	1.21	1.97	5.20	5.85
*Weissella*	6.05	8.15	0.08	0.11
*Bergeyella*	5.15	3.76	0.86	0.54
*Mannheimia*	5.40	4.96	0.48	0.14
*Moraxella*	5.71	3.04	0.01	0
*Pasteurella*	2.56	1.23	1.96	0.49
*Staphylococcus*	2.43	5.21	0.08	0.12
*Lactococcus*	1.25	2.02	1.04	0.38

Similar to what observed for 16S rRNA data, Shannon and Chao1 indexes were significantly higher in nostrils compared to the trachea (Fig. 6B and C). In addition, community composition differed significantly between the two sites (PERMANOVA, *p* = 0.006; Fig. 6D). DESeq2 identified 18 MAGs significantly associated with nostrils (Fig. 6E), including those assigned *Moraxella* (n = 4), *Acinetobacter* (n = 4) and *Staphylococcus* (n = 2). Two MAGs were associated to the trachea, namely *C. perfringens* and *G. parasuis* (Fig. 6E).

### Functional profiling and antimicrobial resistance determinants of MAGs

Using the DRAM pipeline, we identified and annotated 75,345 open reading frames (ORFs) within the 31 MAGs. Among these, 2,820 and 2,083 genes were clustered in Carbohydrate-Active EnZymes (CAZy) families and Clusters of Orthologous Groups (COG) functional categories, respectively (Additional file 1: Table S4). Among CAZy-annotated enzymes, over 70% proteins were classified as either a glycoside hydrolase or a glycosyltransferase, whereas C1 compound metabolism and the electron transport chain formed the core functional categories of the bacterial community, accounting for 44.9% and 27.9% of ORFs, respectively (Additional file 1: Table S4). The distribution of each CAZy families and COG categories among MAGs is provided in Additional file 1: Fig. S5.

Given the high level of genome completeness (87.1–97.3%), the metabolic profile of the four MAGs assigned to the *Moraxella* genus was inspected and compared to the profile previously reported for *Moraxella catarrhalis* ATCC 43617 [33] (Table 3). Interestingly, the metabolic profile inferred from the *Moraxella*-assigned MAGs was comparable to that of *M. catarrhalis* ATCC 43617, with only few differences in the level of completeness of the glyoxylate cycle (Table 3). These results confirm the correct assignment of the four MAGs to the *Moraxella* genus, suggesting that the unclassified MAGs (ID 12, 23 and 30) could represent novel *Moraxella* species.

**Table 3:** Metabolic profiling of metagenome-assembled genomes assigned to the *Moraxella* genus

Species (MAG ID)	Cycle % of completeness				
	*M. porci* (MAG_29)	*Moraxella* unclassified (MAG_12)	*Moraxella* unclassified (MAG_23)	*Moraxella* unclassified (MAG_30)	*M. catarrhalis* ATCC 43617^a^
MAG ID	MAG_29	MAG_12	MAG_23	MAG_30	–
Citrate cycle	87.5	87.5	87.5	100	Almost complete
Entner-Doudoroff pathway	0	0	0	0	Missing
Glycolysis	66. 7	66. 7	66. 7	66. 7	Incomplete
Glyoxylate cycle	100	60.0	60.0	100	Complete
Pentose phosphate cycle	71.4	57.1	71.4	71.4	Incomplete
Reductive acetyl-CoA pathway	28.6	42.9	28.6	42.9	No data
Reductive citrate cycle	50.0	50.0	60.0	60.0	No data
Reductive pentose phosphate cycle	63.6	54.5	63.6	72.7	Incomplete

^a^Data retrieved from ref. [33]

Fifteen antimicrobial resistance genes (ARGs) were identified in the 31 MAGs (Additional file 1: Table S5) and genes conferring resistance to beta-lactams antibiotics (n = 5) and tetracyclines (n = 3) were predominant. Notably, the *mecA* gene was identified in the MAG assigned to *Staphylococcus aureus* (ID 5), which was assigned to a major livestock-associated lineage, *i.e.*, sequence type (ST) 398 by MLST (Additional file 1: Table S5).

## Discussion

This is the first study comparing the bacterial microbiota at different sites of the respiratory tract in pigs. We showed that the microbiota in the choana was similar in community composition to the tracheal microbiota (Fig. 4B), suggesting that the posterior portion of the pig nasal cavity is the primary source of bacteria for the LRT. This observation can be explained by physiological, anatomical and behavioural factors. First, pigs are obligate nasal-breathing species and oral breathing can only occur during pathological conditions. Secondly, the proximity between choana and trachea and the production of nasal secretions is likely to enhance seeding of microorganisms to the LRT by micro-aspiration and mucosal dispersion. Our analysis also revealed a spatial variation in nasal microbial communities, with a higher bacterial diversity and number of differential abundant ASVs in nostrils than in the choana. As previously proposed for humans [34, 35], this difference could reflect persistent bacterial colonization of the posterior nasal cavity compared to transient colonization of the nostrils, which are more exposed to perturbation by inhalation and snout rooting. Due to the presence of dorsal and ventral turbinates, whose function is to heat and filter the inhaled air, the microenvironment of the choana in pigs is less exposed to external perturbations. Altogether, these results are in line with the island model of lung biogeography proposed for humans in ref. [5], according to which the composition of the LRT microbiota is influenced by bacterial migration from the URT.

Bacterial diversity (Fig. 3) and abundance (Additional file 1: Fig. S1) were invariably lower in LRT sites compared to URT sites, which is in line with previous studies in humans and other animal species [3, 4, 36, 37]. Despite the significant difference in community structure between URT and LRT samples (Bray–Curtis dissimilarity; Fig. 4A), a noticeable overlap in microbiota composition was observed between these two biogeographical locations, which is exemplified by the high number of shared genera across all sites (Fig. 1B and Additional file 1: Fig. S2) and of MAGs identified by shotgun sequencing in nasal and tracheal samples (Fig. 6). Overall, *Glassaerella*, *Streptococcus* and *Clostridium* were the predominant genera forming the core pig respiratory microbiome. Differently from previous studies, we employed shotgun metagenomics in combination with 16S rRNA gene sequencing to further understand microbiota composition at the genus and species level in nasal and tracheal samples. Comparison of the most abundant genera identified by 16S rRNA and shotgun sequencing revealed only few discrepancies between the results obtained by these two methods (Table 2), which could be explained by the low resolution of shotgun metagenomics due to host DNA contamination and low bacterial densities. The genome-resolved metagenomic approach we employed reconstructed the draft genomes of most bacterial species associated with abundant ASVs identified by 16S rRNA sequencing (*i.e.*, *C. perfringens*, *M. hyorhinis*, *S. suis* and *B. zoohelcum*). The genus *Rothia* was strongly associated with the nasal cavity, including *Rothia nasimurium*, which has been consistently reported as a common colonizer of the nasal cavities of pigs [38–40]. In addition, an unclassified member of this genus was identified by shotgun sequencing and could represent a novel uncultivated species. Both 16S rRNA and shotgun results confirmed *Moraxella* as one of the dominant genera in the porcine URT, especially in the nose [38, 41, 42]. Notably, three out of the four MAGs assigned to *Moraxella* were putative new species and displayed a metabolic profile similar to that of the human pathogen *M. catarrhalis*. It has recently been speculated that members of this genus might be associated with development of PRDC [38, 43], and that *Moraxella* may exert a protective role by preventing colonization by pathogenic bacteria such as *G. parasuis* [42, 44]. Further research is needed to clarify the possible role of *Moraxella* species in the pathogenesis of PRDC and other diseases in pigs.

Our study shows clear-cut differences in bacterial topography between different sites within the URT or the LRT. For example, the composition of the nasal microbiota differed significantly from that of the tonsillar microbiota (Fig. 4B), and colonization of the URT by different streptococcal species followed a species-specific pattern (Fig. 5). Amongst them, the pathogen *S. suis* was mainly associated with tonsils. This is another original finding of our study since a single previous study investigated both nasal and tonsillar microbiotas in pigs [45] but bacterial composition was resolved up to genus-level only. Although bacterial communities inhabiting different locations within the LRT showed similar composition (Fig. 4B) and diversity (Fig. 3), the genera *Moraxella* and *Streptococcus* were associated to bronchial samples. This observation is likely due to differences related to the sampling methods, *i.e.* lung lavage and bronchial mucosal scraping, and the ability of these bacteria to adhere to the mucosa [46, 47].

Various opportunistic pathogens were detected by our metagenomic analyses. Analysis of 16S rRNA data showed a high abundance of *M. hyorhinis* and *S. suis*, and lower abundance of *P. multocida* and *T. pyogenes.* MAGs assigned to these species except *T. pyogenes* were also identified by shotgun sequencing in nasal and tracheal samples. In addition, *G. parasuis*, the etiological agent of Glässer’s disease, was the most abundant species found by shotgun sequencing, mainly in the trachea. We did not find sequences associated with *A. pleuropneumoniae*, although both herds included in the study were classified in the Danish SPF system as infected with this PRDC pathogen. However, this is not surprising as the common transmission pattern in endemically infected herds entails that this pathogen is mostly be prevalent in the late nursery and finisher stages [48]. At the individual level, *M. hyorhinis* and *G. parasuis* were consistently found in the trachea and nasal cavity of the same pigs, suggesting that nasal carriage of these pathogenic bacteria is a risk factor for LRT colonization.

We acknowledge some limitations of our study. First, the study was limited to six pigs from two Danish farms and therefore the results may not be generalized to the global pig population. In defence of our study, it should be however noted that similar studies in humans [5] and cattle [4] also included low numbers of individuals (8 and 15, respectively) but analysed samples by 16S rRNA sequencing only. Secondly, samples were collected one day after euthanasia and possible alterations in the composition of the respiratory microbiome could have occurred during transportation and storage at refrigeration temperature or as a consequence of contamination occurring during post-mortem sampling, especially for LRT samples, as suggested by the relatively high abundance of enteric bacteria (i.e., *Escherichia-Shigella* and *Clostridium*) in lung aspirate (Fig. 1). Lastly, only a low number of non-host reads were obtained by shotgun sequencing, which induced us to co-assembly data from nostrils and trachea, thereby preventing us from analysing differences in the distribution of the reconstructed MAGs between the two sites. Larger-scale studies on live pigs are needed to confirm the findings of this study and understand how the topography of the bacterial respiratory microbiome varies between individuals, farms and production systems. Since our study focused on young pigs at weaning, longitudinal studies are needed to outline changes in the composition of the respiratory microbiota when older pigs are moved to the finisher barn, where respiratory disease is common. Methods for depletion of host and extracellular DNA should be considered for increasing microbial sequencing depth and consequently the number of species detected by shotgun sequencing [49].

## Conclusions

The study provides new knowledge on microbiota composition and species abundance in distinct ecological niches of the pig respiratory tract using both 16S rRNA and shotgun sequencing. The results shed light on the distribution of opportunistic bacterial pathogens across the respiratory tract and support the hypothesis that bacteria present in the lungs originate from the posterior nasal cavity. Our inability to obtain high resolution profiles using shotgun metagenomics suggests that host DNA depletion is necessary to overcome low bacterial densities and high abundance of host DNA in respiratory samples.

## Supplementary Information

**Additional file 1**: Tables S1–S5 and Figs. S1–S5 reporting statistical results and supplementary microbiome analyses.

## Data Availability

All raw data are publicly available at NCBI’s SRA database under BioProject PRJNA825695.

## Abbreviations

ANI: Average nucleotide identity
ANOSIM: Analysis of similarity
ASV: Amplicon sequence variant
BAL: Bronchoalveolar lavage
CAZy: Carbohydrate-active enzymes
COG: Clusters of orthologous groups
GTDB: Genome taxonomy database
LRT: Lower respiratory tract
MAG: Metagenome-assembled genome
NMDS: Non-metric multi-dimensional scaling
ORF: Open reading frame
PERMANOVA: Permutational multivariate analysis of variance
PRDC: Porcine respiratory diseases complex
URT: Upper respiratory tract
