# Supplementary material for: Bacterial topography of the upper and lower respiratory tract in pigs
Source: Anim Microbiome. 2023 Jan 16;5:5. doi: 10.1186/s42523-023-00226-y (PMC9843957; doi:10.1186/s42523-023-00226-y)
Supplement: Supplementary file 1 — Additional file 1: Tables S1–S5 and Figs. S1–S5 reporting statistical results and supplementary microbiome analyses. [file 42523_2023_226_MOESM1_ESM.docx]

**Bacterial topography of the respiratory tract in pigs**

Mattia Pirolo^a^, Carmen Espinosa-Gongora^a^, Antton Alberdi^b^, Raphael Eisenhofer^b^, Matteo Soverini^c^, Esben Østergaard Eriksen^a^, Ken Steen Pedersen^a^, Luca Guardabassi^a^*

^a^ Department of Veterinary and Animal Sciences, University of Copenhagen, 1870 Frederiksberg C, Denmark

^b^ Center for Evolutionary Hologenomics, GLOBE Institute, University of Copenhagen, 1353 Copenhagen, Denmark

^c^ COPSAC, Copenhagen Prospective Studies on Asthma in Childhood, Herlev and Gentofte Hospital, University of Copenhagen, 2820 Gentofte, Denmark

* Correspondence: Prof. Luca Guardabassi lg@sund.ku.dk

**Table of contents**

| **Table S1.** Wilcoxon rank-sum test results comparing 16S rRNA gene copies and Shannon and Chao1 diversity indexes between sampling locations. | II |
| --- | --- |
| **Table S2.** Pairwise analysis of similarities (ANOSIM) results between sampling sites. | III |
| **Table S3.** Metagenome parameters and taxonomic assignments of 32 metagenome assembled genomes (MAGs) from nasal and tracheal samples. | IV |
| **Table S4.** Summary of the metabolic profile of the porcine respiratory bacterial community. | V |
| **Table S5.** Antimicrobial resistance determinants in metagenome assembled genomes (MAGs) from nasal and tracheal samples. | VI |
| **Figure S1.** Comparison of 16S rRNA gene copies between sampling sites. | VII |
| **Figure S2.** UpSet plot showing shared and unique ASVs in each site. | VIII |
| **Figure S3.** Two-dimensional non-metric multidimensional scaling (nMDS) plot of the microbial community composition in URT and LRT samples. | IX |
| **Figure S4.** Differential abundance analysis between LRT sites. | X |
| **Figure S5.** Relative abundance of Carbohydrate-Active EnZymes (CAZy) families and Clusters of Orthologous Groups (COG) categories in metagenome-assembled genomes (MAGs) assembled from nasal and tracheal samples. | XI |

**Table S1.** Wilcoxon rank-sum test results comparing 16S rRNA gene copies and Shannon and Chao1 diversity indexes between sampling locations.

| **Group 1** | **Group 2** | **16S rRNA gene copies** | | **Shannon index** | | **Chao1 index** | |
| --- | --- | --- | --- | --- | --- | --- | --- |
|  |  | ***p*-value** | ***q*-value** | ***p*-value** | ***q*-value** | ***p*-value** | ***q*-value** |
| Nostrils | Choana | **0.002** | **0.032** | **0.002** | **0.032** | **0.013** | 0.140 |
| Nostrils | Tonsils | 0.310 | 0.620 | 0.818 | 1.000 | 0.937 | 1.000 |
| Nostrils | Trachea | **0.002** | **0.032** | **0.002** | **0.032** | **0.002** | **0.032** |
| Nostrils | Bronchi | **0.004** | **0.048** | 0.310 | 1.000 | **0.041** | 0.290 |
| Nostrils | Lung | **0.002** | **0.032** | **0.015** | 0.150 | **0.009** | 0.100 |
| Choana | Tonsils | **0.004** | **0.048** | **0.002** | **0.032** | **0.020** | 0.180 |
| Choana | Trachea | 0.093 | 0.370 | 1.000 | 1.000 | 0.936 | 1.000 |
| Choana | Bronchi | 0.394 | 0.620 | **0.004** | **0.048** | 0.128 | 0.640 |
| Choana | Lung | **0.015** | 0.110 | 0.394 | 1.000 | 0.470 | 1.000 |
| Tonsils | Trachea | **0.026** | 0.160 | **0.002** | **0.032** | **0.002** | **0.032** |
| Tonsils | Bronchi | **0.009** | 0.069 | 0.093 | 0.560 | **0.026** | 0.210 |
| Tonsils | Lung | **0.002** | **0.032** | **0.015** | 0.150 | **0.004** | 0.056 |
| Trachea | Bronchi | 0.065 | 0.320 | **0.026** | 0.210 | **0.015** | 0.150 |
| Trachea | Lung | **0.004** | **0.048** | 0.699 | 1.000 | 0.521 | 1.000 |
| Bronchi | Lung | 0.180 | 0.540 | **0.041** | 0.290 | **0.041** | 0.290 |

*p*-values were corrected for multiple comparisons using Holm’s correction. *p*-values and corrected *p*-values (*q*-values) > 0.05 are highlighted in bold.

**Table S2.** Pairwise analysis of similarities (ANOSIM) results between sampling sites.

| **Group 1** | **Group 2** | **R-value** | ***p*-value** | ***q*-value** |
| --- | --- | --- | --- | --- |
| Nostrils | Choana | 0.37 | **0.015** | **0.030** |
| Nostrils | Tonsils | 0.85 | **0.003** | **0.019** |
| Nostrils | Trachea | 0.46 | **0.007** | **0.021** |
| Nostrils | Bronchi | 0.37 | **0.026** | **0.033** |
| Nostrils | Lung | 0.40 | **0.019** | **0.030** |
| Choana | Tonsils | 0.93 | **0.003** | **0.019** |
| Choana | Trachea | 0.24 | 0.073 | 0.084 |
| Choana | Bronchi | 0.53 | **0.015** | **0.030** |
| Choana | Lung | 0.31 | **0.026** | **0.033** |
| Tonsils | Trachea | 0.42 | **0.005** | **0.019** |
| Tonsils | Bronchi | 0.67 | **0.005** | **0.019** |
| Tonsils | Lung | 0.40 | **0.018** | **0.030** |
| Trachea | Bronchi | 0.34 | **0.020** | **0.030** |
| Trachea | Lung | 0.10 | 0.930 | 0.930 |
| Bronchi | Lung | 0.17 | 0.712 | 0.763 |

*p*-values were corrected for multiple comparisons using Benjamini-Hochberg’s correction. *p*-values and corrected *p*-values (*q*-values) > 0.05 are highlighted in bold. R-values indicate the strength of the dissimilarities, where 1 is the strongest and 0 is weakest.

**Table S3.** Metagenome parameters and taxonomic assignments of 32 metagenome assembled genomes (MAGs) from nasal and tracheal samples.

| **Bin** | **Completeness (%)** | **Contamination (%)** | **GC content (%)** | **Contigs (no.)** | **N50** | **Lenght (bp)** | **tRNA (no.)** | **Reference** | **ANI^a^** | **Phylum** | **Class** | **Order** | **Family** | **Genus** | **Species** |
| --- | --- | --- | --- | --- | --- | --- | --- | --- | --- | --- | --- | --- | --- | --- | --- |
| MAG_28 | 100.0 | 0.0 | 32.6 | 18 | 370101 | 3,590,969 | 72 | GCF_000382425.1 | 98.3 | *Bacteroidetes* | *Bacteroidia* | *Flavobacteriales* | *Weeksellaceae* | *Empedobacter* | *E. brevis* |
| MAG_22 | 100.0 | 0.2 | 35.7 | 109 | 37306 | 2,509,448 | 36 | GCF_000301075.1 | 97.5 | *Bacteroidetes* | *Bacteroidia* | *Flavobacteriales* | *Weeksellaceae* | *Bergeyella* | *B. zoohelcum* |
| MAG_20 | 100.0 | 0.0 | 25.8 | 22 | 64559 | 831,966 | 29 | GCF_000383515.1 | 99.6 | *Firmicutes* | *Bacilli* | *Mycoplasmatales* | *Metamycoplasmataceae* | *Mesomycoplasma* | *M. hyorhinis* |
| MAG_10 | 100.0 | 0.5 | 38.9 | 59 | 100192 | 2,883,521 | 36 | GCF_002205795.1 | 96.0 | *Bacteroidetes* | *Bacteroidia* | *Flavobacteriales* | *Weeksellaceae* | *Kaistella* | *Kaistella spp.* |
| MAG_21 | 99.8 | 0.0 | 40.2 | 12 | 234973 | 2,136,558 | 53 | GCF_013377235.1 | 96.9 | *Proteobacteria* | *Gammaproteobacteria* | *Enterobacterales* | *Pasteurellaceae* | *Mannheimia* | *M. varigena* |
| MAG_07 | 99.5 | 1.7 | 42.2 | 68 | 97334 | 3,612,486 | 60 | GCF_002165375.2 | 97.2 | *Proteobacteria* | *Gammaproteobacteria* | *Pseudomonadales* | *Moraxellaceae* | *Acinetobacter* | *A. chinensis* |
| MAG_11 | 98.8 | 0.0 | 39.9 | 78 | 43560 | 1,942,703 | 26 | GCF_000420785.1 | 97.7 | *Firmicutes* | *Bacilli* | *Lactobacillales* | *Streptococcaceae* | *Streptococcus* | *S. hyovaginalis* |
| MAG_04 | 98.6 | 0.5 | 47.3 | 112 | 28940 | 2,077,083 | 42 | - | - | *Proteobacteria* | *Gammaproteobacteria* | *Burkholderiales* | *Neisseriaceae* | *-* | *-* |
| MAG_08 | 98.5 | 1.8 | 44.4 | 267 | 14954 | 2,700,654 | 77 | GCF_000193635.1 | 96.6 | *Firmicutes* | *Bacilli* | *Lactobacillales* | *Lactobacillaceae* | *Weissella* | *W. cibaria* |
| MAG_15 | 98.0 | 1.0 | 40.2 | 193 | 14657 | 2,213,163 | 48 | GCF_000754275.1 | 98.5 | *Proteobacteria* | *Gammaproteobacteria* | *Enterobacterales* | *Pasteurellaceae* | *Pasteurella* | *P. multocida* |
| MAG_29 | 97.3 | 0.8 | 46.7 | 101 | 30709 | 2,143,341 | 43 | GCF_002014855.1 | 96.3 | *Proteobacteria* | *Gammaproteobacteria* | *Pseudomonadales* | *Moraxellaceae* | *Moraxella* | *M. porci* |
| MAG_13 | 97.3 | 0.6 | 50.6 | 232 | 34341 | 5,094,003 | 81 | GCF_002950215.1 | 97.8 | *Proteobacteria* | *Gammaproteobacteria* | *Enterobacterales* | *Enterobacteriaceae* | *Shigella* | *S. flexneri* |
| MAG_09 | 96.8 | 1.4 | 39.7 | 200 | 20650 | 2,029,694 | 46 | GCF_002015085.1 | 97.1 | *Proteobacteria* | *Gammaproteobacteria* | *Enterobacterales* | *Pasteurellaceae* | *Glaesserella* | *G. parasuis* |
| MAG_17 | 96.3 | 0.0 | 38.3 | 69 | 51883 | 2,294,895 | 23 | GCF_000380145.1 | 99.5 | *Firmicutes* | *Bacilli* | *Lactobacillales* | *Streptococcaceae* | *Streptococcus* | *S. thoraltensis* |
| MAG_01 | 96.1 | 0.1 | 43.8 | 108 | 24105 | 1,953,507 | 14 | GCF_002962445.1 | 95.4 | *Firmicutes* | *Bacilli* | *Lactobacillales* | *Streptococcaceae* | *Streptococcus* | *S. suis* |
| MAG_18 | 95.8 | 1.3 | 50.4 | 217 | 13885 | 2,373,918 | 43 | - | - | *Bacteroidetes* | *Bacteroidia* | *Bacteroidales* | *Porphyromonadaceae* | *Porphyromonas* | *-* |
| MAG_05 | 95.7 | 6.1 | 32.7 | 141 | 31614 | 2,810,197 | 35 | GCF_001027105.1 | 97.5 | *Firmicutes* | *Bacilli* | *Staphylococcales* | *Staphylococcaceae* | *Staphylococcus* | *S. aureus* |
| MAG_30 | 95.6 | 0.4 | 43.6 | 109 | 29500 | 2,386,675 | 40 | - | - | *Proteobacteria* | *Gammaproteobacteria* | *Pseudomonadales* | *Moraxellaceae* | *Moraxella* | *-* |
| MAG_25 | 94.7 | 1.4 | 35.9 | 87 | 42525 | 2,149,811 | 50 | GCF_000816085.1 | 99.6 | *Firmicutes* | *Bacilli* | *Staphylococcales* | *Staphylococcaceae* | *Staphylococcus* | *S. hyicus* |
| MAG_23 | 94.2 | 1.1 | 43.6 | 83 | 36131 | 2,032,057 | 41 | - | - | *Proteobacteria* | *Gammaproteobacteria* | *Pseudomonadales* | *Moraxellaceae* | *Moraxella* | *-* |
| MAG_06 | 94.1 | 1.4 | 34.9 | 251 | 12712 | 2,301,371 | 45 | GCF_900099625.1 | 98.8 | *Firmicutes* | *Bacilli* | *Lactobacillales* | *Streptococcaceae* | *Lactococcus* | *L. lactis* |
| MAG_02 | 89.9 | 3.0 | 38.5 | 353 | 4765 | 1,460,952 | 42 | GCF_002706375.1 | 97.7 | *Firmicutes* | *Bacilli* | *Lactobacillales* | *Lactobacillaceae* | *Lactobacillus* | *L. amylovorus* |
| MAG_24 | 87.9 | 7.1 | 60.2 | 177 | 22602 | 2,355,338 | 54 | - | - | *Actinobacteria* | *Actinomycetia* | *Actinomycetales* | *Micrococcaceae* | *Rothia* | *-* |
| MAG_14 | 87.4 | 3.1 | 58 | 1204 | 3898 | 4,229,998 | 48 | GCF_000742135.1 | 99.2 | *Proteobacteria* | *Gammaproteobacteria* | *Enterobacterales* | *Enterobacteriaceae* | *Klebsiella* | *K. pneumoniae* |
| MAG_12 | 87.1 | 1.1 | 41.3 | 203 | 15012 | 2,105,558 | 36 | - | - | *Proteobacteria* | *Gammaproteobacteria* | *Pseudomonadales* | *Moraxellaceae* | *Moraxella* | *-* |
| MAG_27 | 86.1 | 2.3 | 37.8 | 245 | 21768 | 3,979,153 | 42 | GCF_000368925.1 | 97.8 | *Proteobacteria* | *Gammaproteobacteria* | *Pseudomonadales* | *Moraxellaceae* | *Acinetobacter* | *A. bereziniae* |
| MAG_03 | 83.7 | 0.8 | 33.5 | 286 | 4672 | 1,246,547 | 4 | - | - | *Firmicutes* | *Bacilli* | *Lactobacillales* | *Aerococcaceae* | *-* | *-* |
| MAG_16 | 83.6 | 3.5 | 27.9 | 823 | 3525 | 2,855,497 | 61 | GCF_000013285.1 | 97.4 | *Firmicutes* | *Clostridia* | *Clostridiales* | *Clostridiaceae* | *Clostridium* | *C. perfringens* |
| MAG_26 | 83.4 | 1.7 | 39.8 | 618 | 4643 | 2,464,488 | 64 | GCF_001678755.1 | 99.1 | *Proteobacteria* | *Gammaproteobacteria* | *Pseudomonadales* | *Moraxellaceae* | *Acinetobacter* | *A. gandensis* |
| MAG_31 | 79.3 | 4.8 | 41.2 | 532 | 4844 | 2,231,337 | 25 | GCF_003711395.1 | 99.6 | *Proteobacteria* | *Gammaproteobacteria* | *Pseudomonadales* | *Moraxellaceae* | *Acinetobacter* | *Acinetobacter spp.* |
| MAG_32 | 73.0 | 4.3 | 37.1 | 489 | 3956 | 1,888,389 | 23 | GCF_003143955.1 | 98.2 | *Firmicutes* | *Bacilli* | *Bacillales* | *Planococcaceae* | *Kurthia* | *K. zopfii* |
| MAG_19 | 71.9 | 1.0 | 33 | 463 | 4025 | 1,767,022 | 3 | GCF_009695815.1 | 97.7 | *Firmicutes* | *Clostridia* | *Peptostreptococcales* | *Peptostreptococcaceae* | *Peptostreptococcus* | *P. anaerobius* |

^a^Average Nucletide identity

**Table S4.** Summary of the metabolic profile of the porcine respiratory bacterial community.

| **Enzyme categories** | **No. of genes (%)** |
| --- | --- |
| **CAZy family** |  |
| Glycoside hydrolase | 1,084 (38.0) |
| Glycosyltransferase | 1,043 (36.6) |
| Carbohydrate-binding module | 282 (9.9) |
| Carbohydrate esterase | 272 (9.5) |
| Auxiliary activitie | 137 (4.8) |
| Polysaccharide lyase | 32 (1.1) |
| **COG functional category** |  |
| C_1_ compound metabolism | 935 (44.9) |
| Electron transport chain | 581 (27.9) |
| Oxygen metabolism | 192 (9.2) |
| Nitrogen metabolism | 127 (6.1) |
| C_1_-methane metabolism | 83 (4.0) |
| Sulfur metabolism | 65 (3.1) |
| Metal reduction | 26 (1.2) |
| Other metabolisms | 74 (3.6) |

CAZy, Carbohydrate-Active EnZymes; COG, Clusters of Orthologous Groups.

**Table S5.** Antimicrobial resistance determinants in metagenome assembled genomes (MAGs) from nasal and tracheal samples.

| Antimicrobial class | Gene | *Acinetobacter bereziniae*  (MAG_27) | *Clostridium perfringens*  (MAG_16) | *Empedobacter brevis*  (MAG_28) | *Klebsiella pneumoniae*  (MAG_14) | *Moraxella* unclassified  (MAG_30) | *Peptostreptococcus anaerobius*  (MAG_19) | *Staphylococcus aureus*  (MAG_5) | *Streptococcus hyovaginalis*  (MAG_11) |
| --- | --- | --- | --- | --- | --- | --- | --- | --- | --- |
| Aminoglycoside | *ant(6)-Ia* |  |  |  |  |  |  |  | X |
| Beta-lactam | *blaBRO-1* |  |  |  |  | X |  |  |  |
| Beta-lactam | *blaEBR-1* |  |  | X |  |  |  |  |  |
| Beta-lactam | *blaOXA-355* | X |  |  |  |  |  |  |  |
| Beta-lactam | *blaSHV-11* |  |  |  | X |  |  |  |  |
| Beta-lactam | *mecA* |  |  |  |  |  |  | X |  |
| Fosfomycin | *fosA* |  |  |  | X |  |  |  |  |
| Lincosamide | *lnu*(B) |  |  |  |  |  |  |  | X |
| Lincosamide | *lsa*(E) |  |  |  |  |  |  |  | X |
| Macrolides | *erm*(47) |  |  |  |  |  | X |  |  |
| Quinolones | *oqxB* |  |  |  | X |  |  |  |  |
| Tetracycline | *tet*(A) |  | X |  |  |  |  |  |  |
| Tetracycline | *tet*(H) |  |  |  |  | X |  |  |  |
| Tetracycline | *tet*(T) |  |  |  |  |  | X |  |  |
| Zinc/cadmium | *czrC* |  |  |  |  |  |  | X |  |


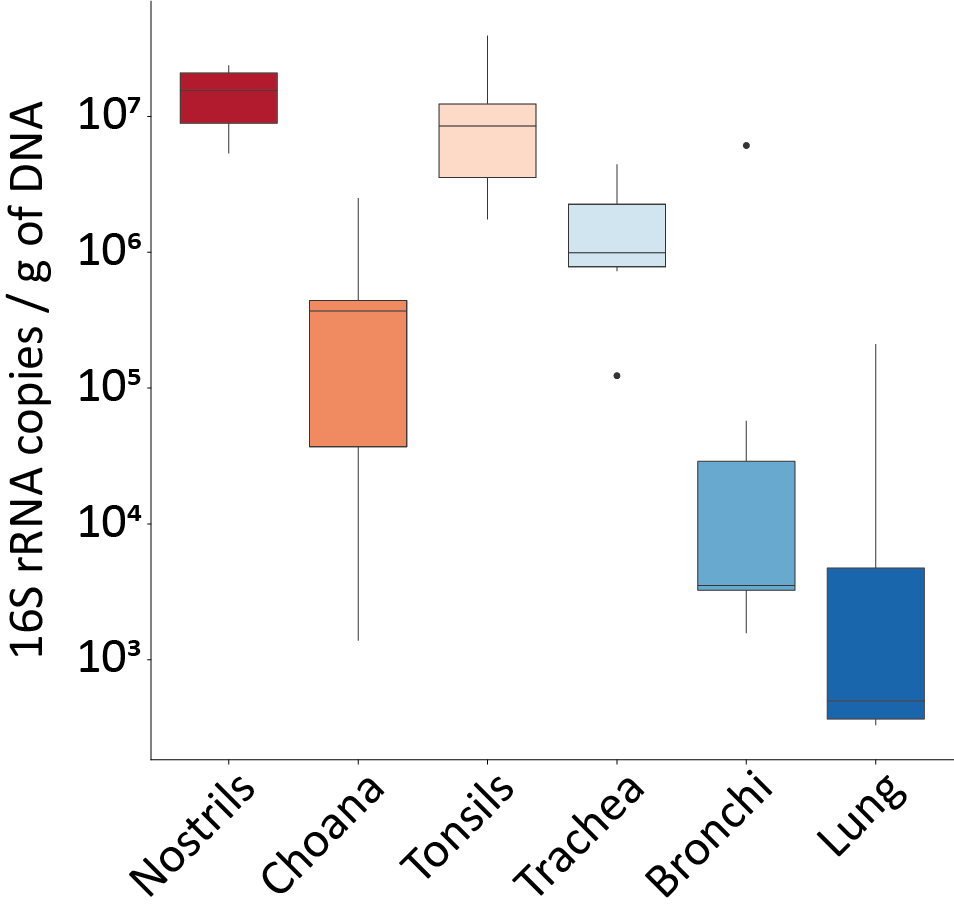


**Fig. S1.** Comparison of 16S rRNA gene copies between sampling sites.


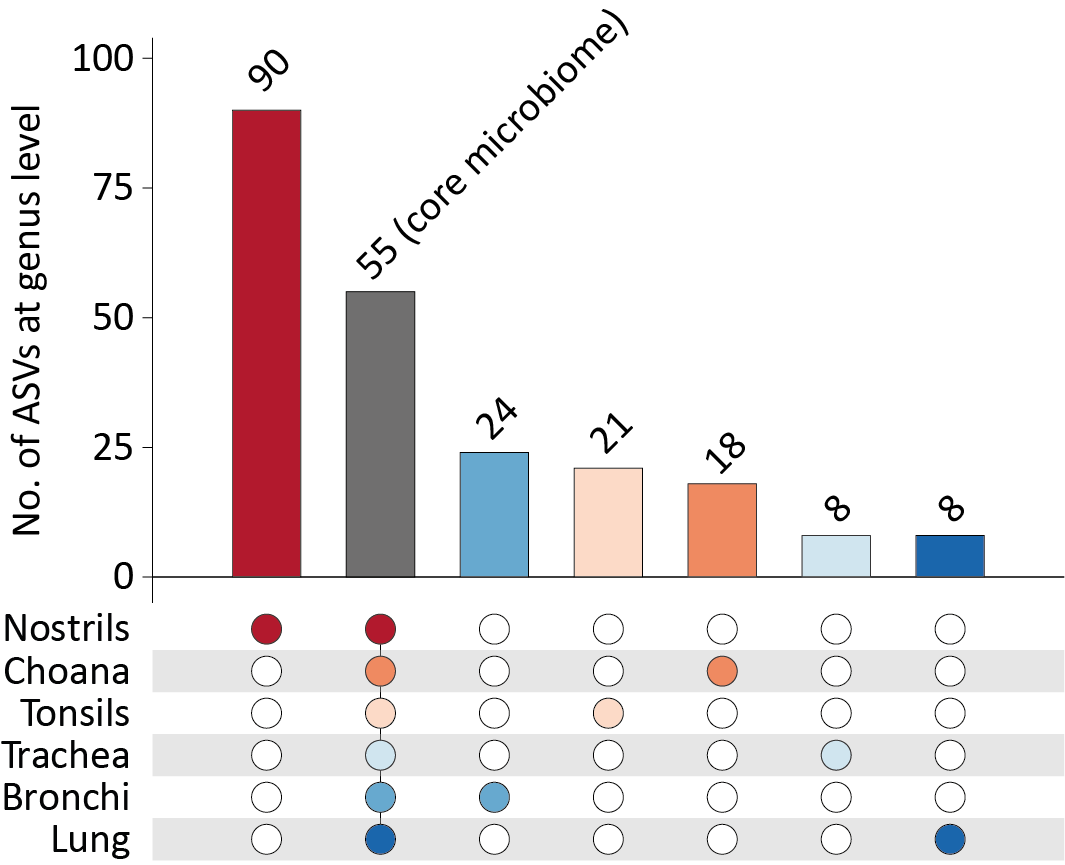


**Fig. S2.** UpSet plot showing shared and unique ASVs in each site. Calculations are based on ASV table agglomerated at genus level. The histogram shows the number of unique and shared genera at each circle or intersection, respectively. Connected circles in the matrix indicate shared genera between sampling locations. Unconnected circles denote individual site and represent unique genera in each location.


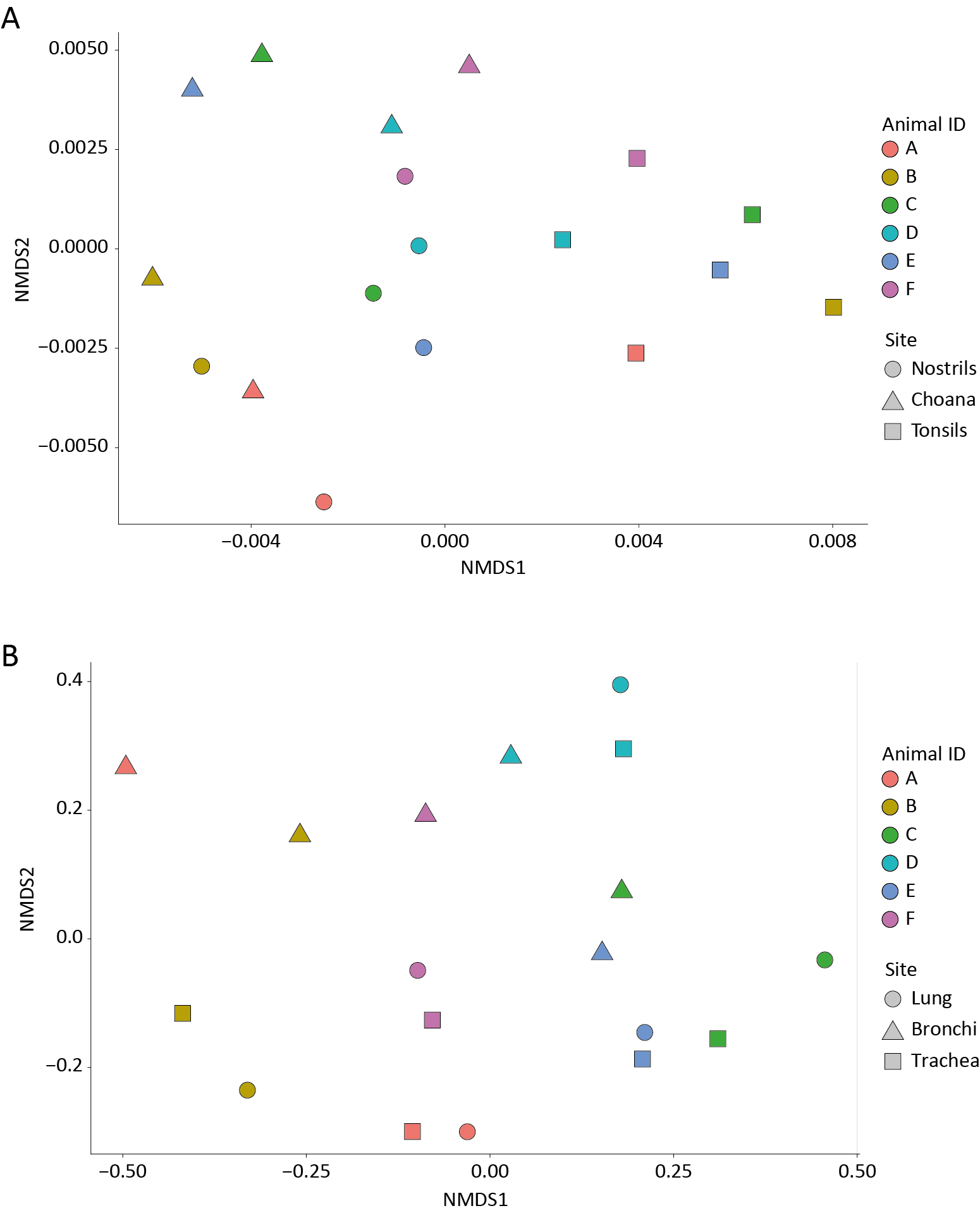


**Fig. S3.** Two-dimensional non-metric multidimensional scaling (nMDS) plot of the microbial community composition in URT (A) and LRT (B) samples.


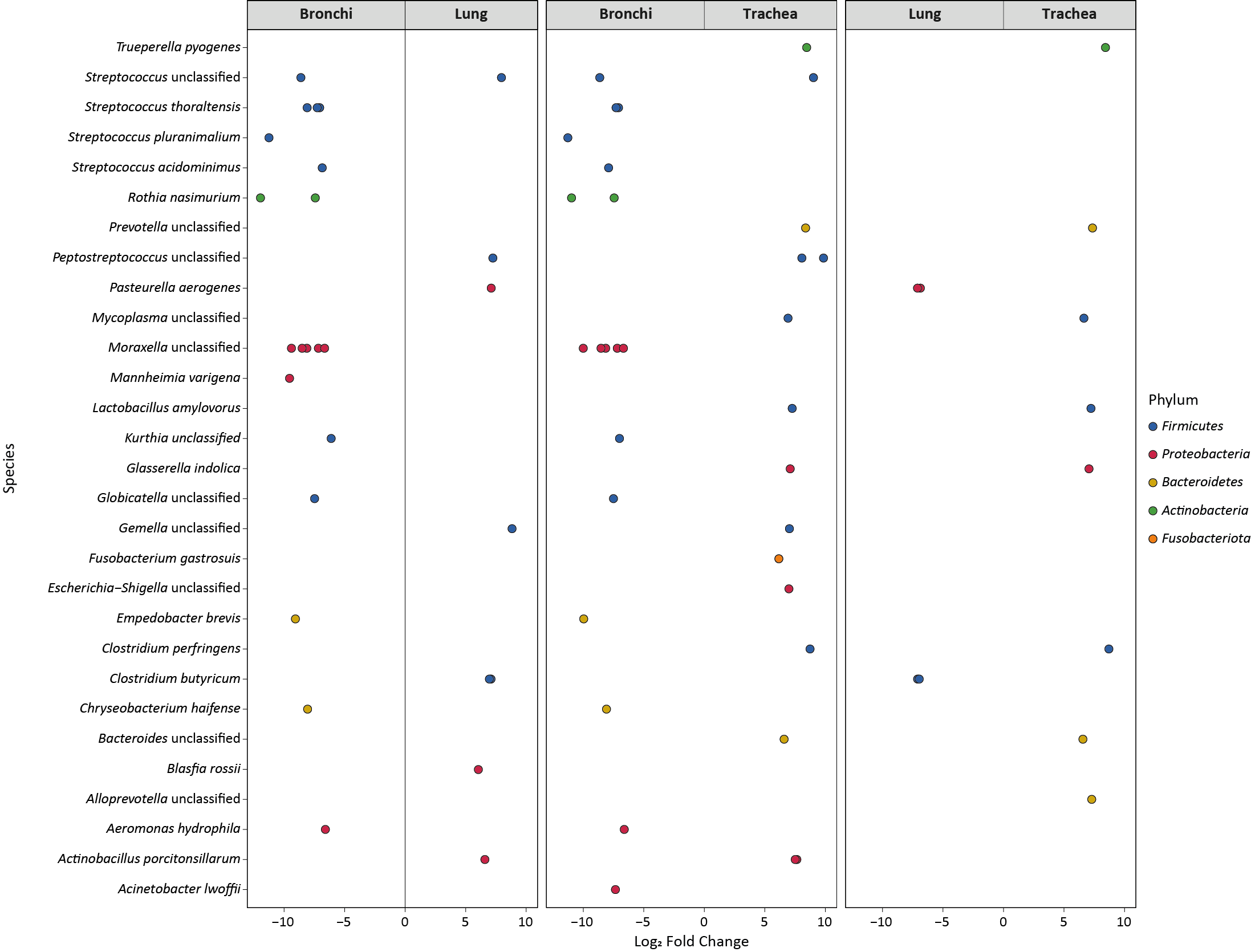


**Fig. S4.** Differential abundance analysis between LRT sites. ASVs were identified to be of significantly differential abundance by DESeq2. Only ASVs with *q*-values (adjusted *p*-values) < 0.01, estimated fold change > 6 or < -6, and estimated base mean > 60 were considered significantly differentially abundant and included in the plot.


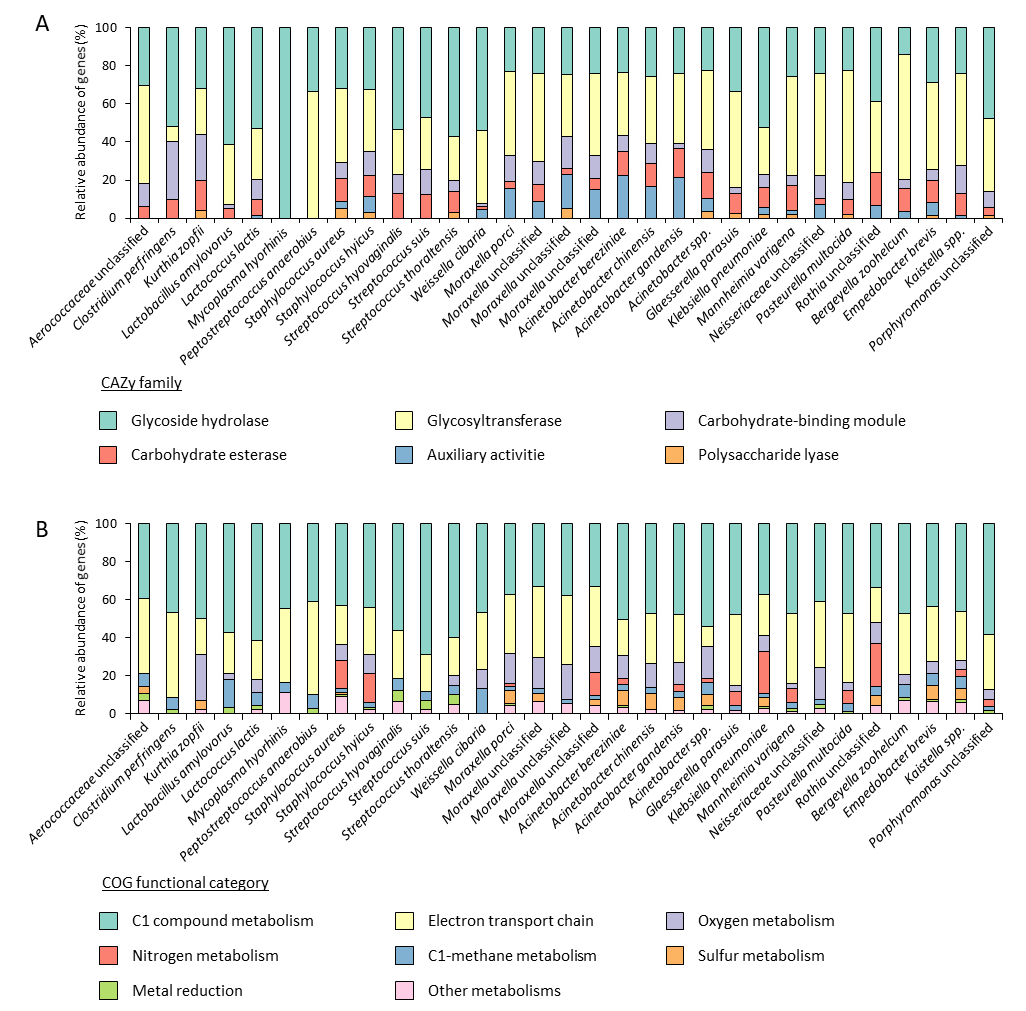


**Fig. S5.** Relative abundance of Carbohydrate-Active EnZymes (CAZy) families (A) and Clusters of Orthologous Groups (COG) categories (B) in metagenome-assembled genomes (MAGs) from nasal and tracheal samples.
